# Craniosynostosis surgery: workflow based on virtual surgical planning, intraoperative navigation and 3D printed patient-specific guides and templates

**DOI:** 10.1038/s41598-019-54148-4

**Published:** 2019-11-27

**Authors:** David García-Mato, Santiago Ochandiano, Mónica García-Sevilla, Carlos Navarro-Cuéllar, Juan V. Darriba-Allés, Roberto García-Leal, José A. Calvo-Haro, Rubén Pérez-Mañanes, José I. Salmerón, Javier Pascau

**Affiliations:** 1 Departamento de Bioingeniería e Ingeniería Aeroespacial, Universidad Carlos III de Madrid, Madrid, Spain; 2 Instituto de Investigación Sanitaria Gregorio Marañón, Madrid, Spain; 3 Servicio de Cirugía Oral y Maxilofacial, Hospital General Universitario Gregorio Marañón, Madrid, Spain; 4 Servicio de Neurocirugía, Hospital General Universitario Gregorio Marañón, Madrid, Spain; 5 Servicio de Cirugía Ortopédica y Traumatología, Hospital General Universitario Gregorio Marañón, Madrid, Spain

**Keywords:** Three-dimensional imaging, Paediatrics, Biomedical engineering

## Abstract

Craniosynostosis must often be corrected using surgery, by which the affected bone tissue is remodeled. Nowadays, surgical reconstruction relies mostly on the subjective judgement of the surgeon to best restore normal skull shape, since remodeled bone is manually placed and fixed. Slight variations can compromise the cosmetic outcome. The objective of this study was to describe and evaluate a novel workflow for patient-specific correction of craniosynostosis based on intraoperative navigation and 3D printing. The workflow was followed in five patients with craniosynostosis. Virtual surgical planning was performed, and patient-specific cutting guides and templates were designed and manufactured. These guides and templates were used to control osteotomies and bone remodeling. An intraoperative navigation system based on optical tracking made it possible to follow preoperative virtual planning in the operating room through real-time positioning and 3D visualization. Navigation accuracy was estimated using intraoperative surface scanning as the gold-standard. An average error of 0.62 mm and 0.64 mm was obtained in the remodeled frontal region and supraorbital bar, respectively. Intraoperative navigation is an accurate and reproducible technique for correction of craniosynostosis that enables optimal translation of the preoperative plan to the operating room.

## Introduction

Craniosynostosis is a congenital defect that is defined as the premature fusion of one or more cranial vault sutures^1^. According to recent prevalence studies, this condition affects approximately one in 2000–2500 live births worldwide^2–4^. Craniosynostosis usually occurs as an isolated event (*nonsyndromic craniosynostosis*), although it can also be associated with a specific syndrome (*syndromic craniosynostosis*).

Morphological abnormalities associated with craniosynostosis include dysmorphic cranial vault and facial asymmetry. According to Virchow’s law, calvarial growth occurs in a plane parallel to that of the fused suture, while growth in the perpendicular plane is disrupted^5^. Consequently, deformations of the calvaria provide information about which sutures are fused. Therefore, craniosynostosis can be classified in terms of the affected sutures and the resulting malformation as follows: sagittal (scaphocephaly), metopic (trigonocephaly), coronal (anterior plagiocephaly), and lambdoid (posterior plagiocephaly)^6^. These morphological abnormalities may also have functional consequences, such as elevated intracranial pressure and impaired brain growth^7^.

Although significant improvements have been made in the management of craniosynostosis, surgical management is the preferred treatment in most cases. Minimally invasive techniques have been proposed^8^, but these approaches are typically reserved for patients less than 6 months old with mild malformations affecting only one suture^9^. Most commonly accepted methods of treatment include open cranial vault remodeling to normalize the calvarial shape with the aim of increasing intracranial volume, thus reducing the risk of elevated intracranial pressure. Surgery consists of three steps: (1) removal of the affected bones, (2) remodeling of the bones into the most appropriate shape for the patient, and (3) placement and fixation of remodeled bones^10^. The consensus is to operate on patients before the first year of life in order to maximize reossification by taking advantage of bone malleability during this early age^11^.

Nowadays, diagnosis and surgical correction of craniosynostosis rely mostly on the subjective judgement of the surgeon, who determines the degree of the deformity and the approach to remodeling the affected bone to best restore normal skull shape. This approach usually increases the duration of surgery and is highly dependent on the experience of the surgeon. Computer-assisted surgical planning has been proposed to improve the accuracy and efficiency of these surgical procedures^12,13^. Thus, osteotomies can be designed preoperatively, and bones can be virtually configured to achieve the desired shape and features. Normative skull models could be used as a reference during virtual cranial vault reshaping considering the age and gender of the patient for more objective planning^14–16^. In addition, surgeries can be simulated preoperatively using realistic 3D printed skull models to improve treatment quality and medical training^17–19^.

In addition, computer-aided design and manufacturing (CAD/CAM) enables the generation of cutting guides and shaping templates adapted to the patient’s bone, thus allowing surgeons to transfer preoperative virtual planning into craniosynostosis surgery and improving the accuracy of the procedure^13,20–22^. Once osteotomy is performed, bone is resected, and open cranial vault reconstruction is performed using the templates for guidance. Finally, the remodeled bone is manually placed and fixed. However, the exact placement of the reshaped bone tissue is assessed visually, and the final position may differ from that planned preoperatively. Slight positional or rotational variations in the location of the bone can compromise symmetry, harmony, and balance between the face and the cranial vault and, therefore, the cosmetic outcome.

In this context, image-guided surgery (IGS) technology can be used to assist during bone fragment positioning. IGS enables accurate real-time positioning of surgical instruments and devices with respect to the patient’s anatomy. This technology has been used in many fields to improve the performance, speed, and safety of surgical procedures^23^. IGS based on real-time tool tracking has not yet been applied for craniosynostosis surgery owing to its invasiveness and discomfort associated with head immobilization and the attachment of landmarks for intraoperative registration. Moreover, head immobilization is rarely used in patients under 2 years of age due to potential risk of skull and brain injury from pin fixation. Kobets *et al*.^24^ proposed the use of intraoperative computed tomography (CT) imaging to assess remodeled bone placement. However, as this methodology requires the infant to be exposed to ionizing radiation and does not enable real-time positioning, it limits the ability to make the adjustments necessary to achieve an optimal outcome.

In this study, we propose a new workflow for surgical correction of craniosynostosis based on intraoperative navigation and patient-specific 3D printed guides and templates. Our objective was to evaluate IGS for positioning of remodeled bone in craniosynostosis surgery. The proposed workflow was followed in five patients affected by common types of single-suture synostosis. Our workflow does not require head immobilization or preoperative attachment of registration landmarks, thus avoiding invasiveness and patient discomfort. To our knowledge, this is the first study to apply intraoperative navigation with IGS technology for the correction of craniosynostosis.

## Methods

**Figure 1:**
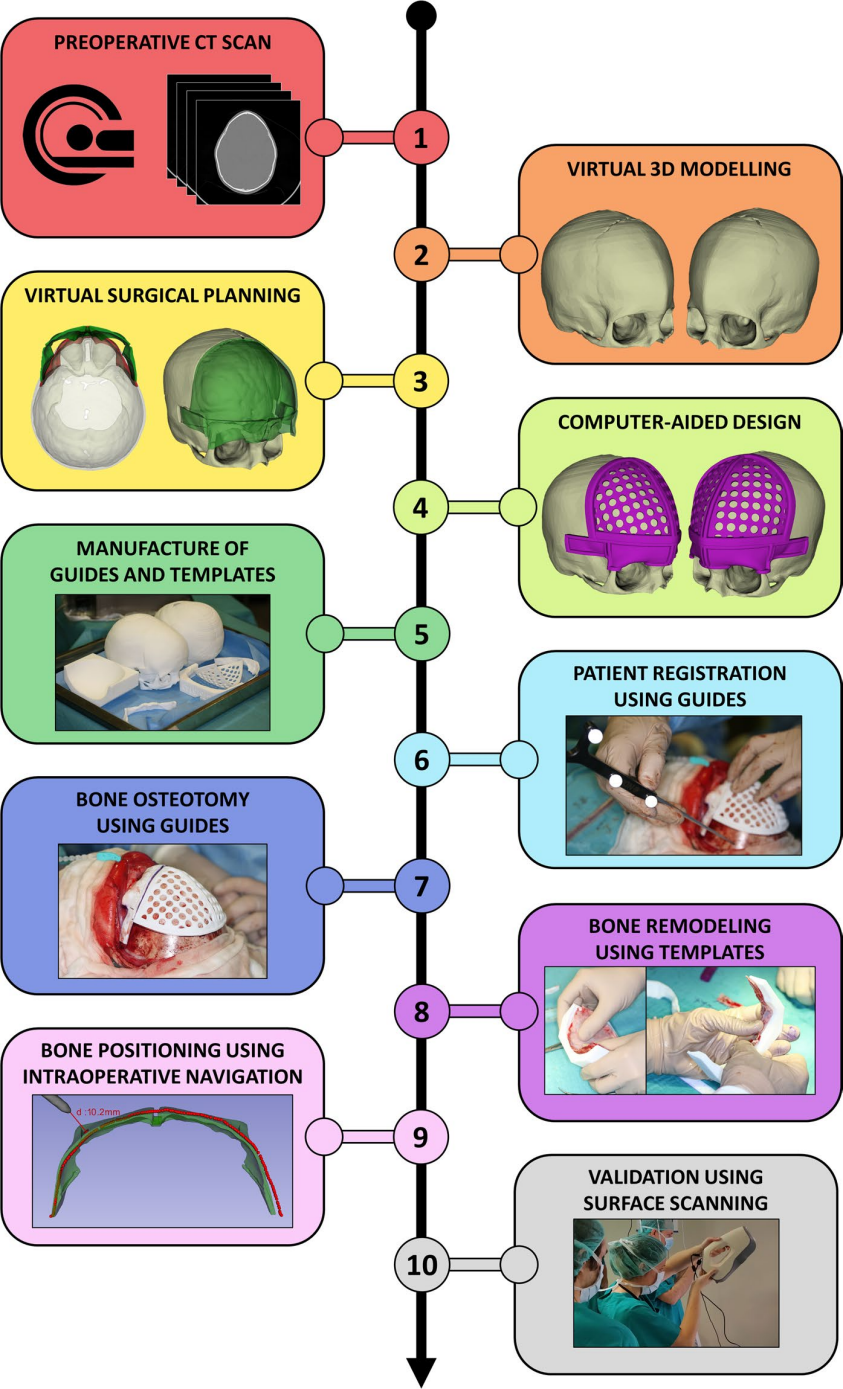
Proposed workflow for surgical correction of craniosynostosis.

We first describe the subjects included in this study. Then, we present the methodology used to design and manufacture patient-specific guides and templates for osteotomy and remodeling. Next, we describe the intraoperative navigation system and surgical procedure followed for remodeled bone positioning. Finally, an intraoperative surface scanning technique and methods for data analysis and evaluation are detailed. A summary of the proposed framework is presented in Fig. 1.

### Cases

The proposed workflow was followed in five patients: an 8-month-old girl with trigonocephaly, a 9-month-old boy with trigonocephaly, a 13-month-old girl with anterior plagiocephaly, a 15-month-old (corrected age is 12 months) girl with trigonocephaly and a 16-month-old (corrected age is 13 months) boy with trigonocephaly. The patients are nonsyndromic and had not undergone prior craniofacial surgeries and did not present hydrocephalus, intracranial hemorrhages, or craniofacial trauma. The parent or legal guardian of the patients signed an informed consent for study participation and a specific consent for publication of identifying images (patient 1). The study was performed in accordance with the principles of the 1964 Declaration of Helsinki as revised in 2013 and was approved by the Research Ethics Committee at Hospital General Universitario Gregorio Marañón.

### CT image acquisition and processing

**Figure 2:**
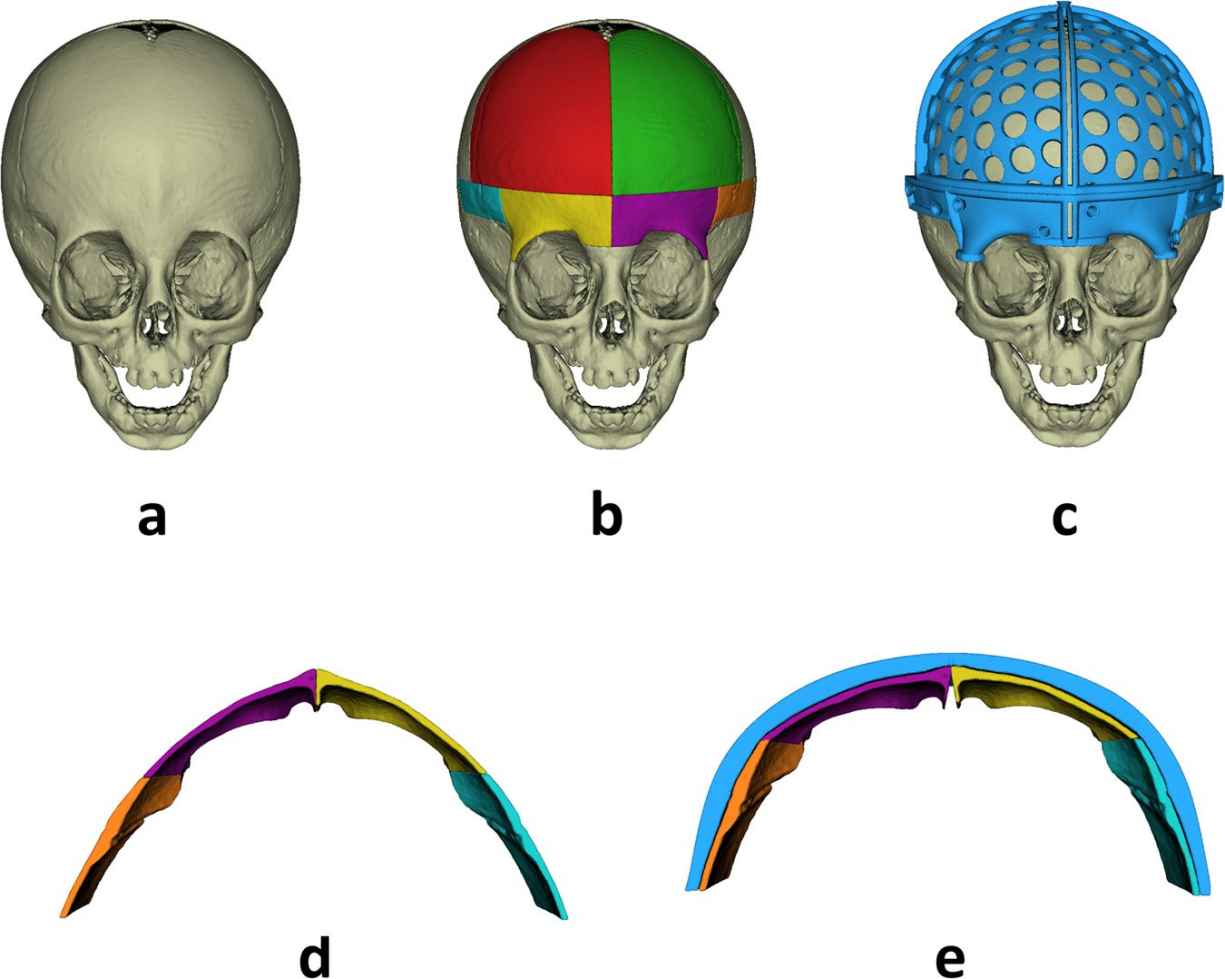
Virtual surgical planning: (**a**) preoperative skull model, (**b**) planned osteotomies, (**c**) designed cutting guides, (**d**) preoperative supraorbital bar, (**e**) remodeled supraorbital bar using patient-specific template.

A preoperative cranial CT scan was acquired for all patients with a Philips Mx8000 scanner. The axial in-plane pixel size was 0.25 mm, and the slice thickness was 1.3 mm for all cases. CT imaging is the standard procedure in our center for confirming the diagnosis by evaluating the state of cranial sutures. Bone tissue was segmented from CT images with intensity-based algorithms, and a 3D model of the skull was generated on 3D Slicer software^25^. The model was postprocessed to eliminate unnecessary small-scale anatomical structures not 3D-connected with the region of interest. Then, the segmented model was smoothed using a Laplacian smoothing algorithm (iterations = 10, relaxation factor = 0.1) to remove stair-step artifact. Holes in the output mesh were filled to ensure continuity in the final mesh. The Surface Toolbox in 3D Slicer software was used in all postprocessing steps. The final continuous 3D mesh was used as a reference for computer-assisted preoperative planning (Fig. 2a).

### Virtual surgical planning and computer-aided design

**Figure 3:**
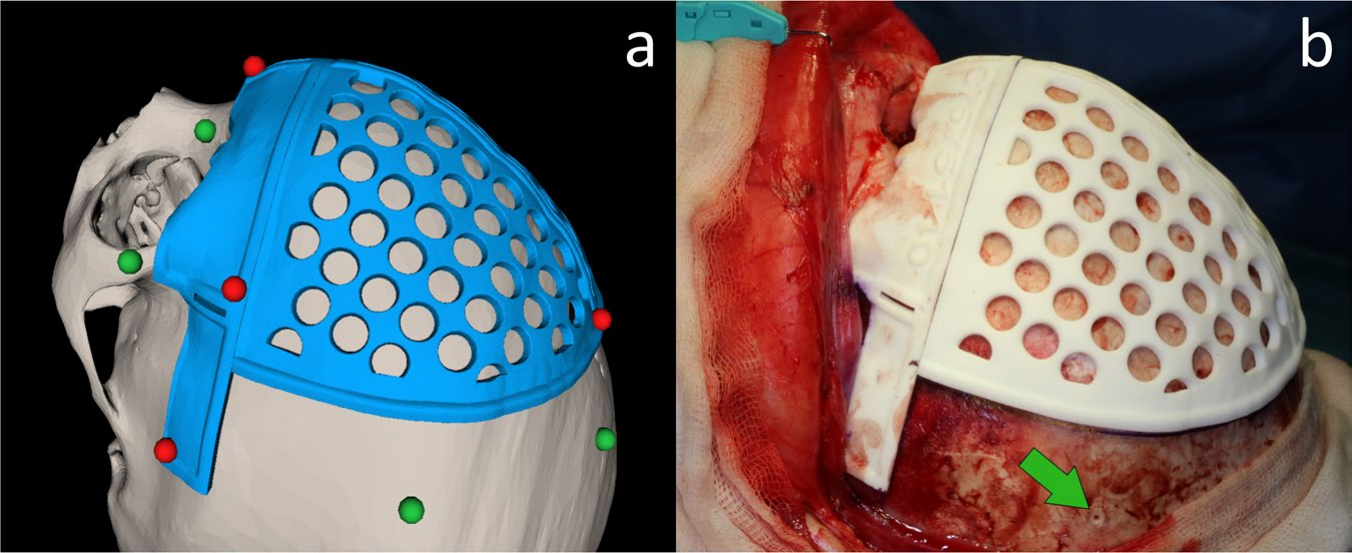
(**a**) Virtual models and distribution of registration landmarks for primary (red) and secondary registration (green). (**b**) Resorbable pin attached intraoperatively for secondary registration.

**Figure 4:**
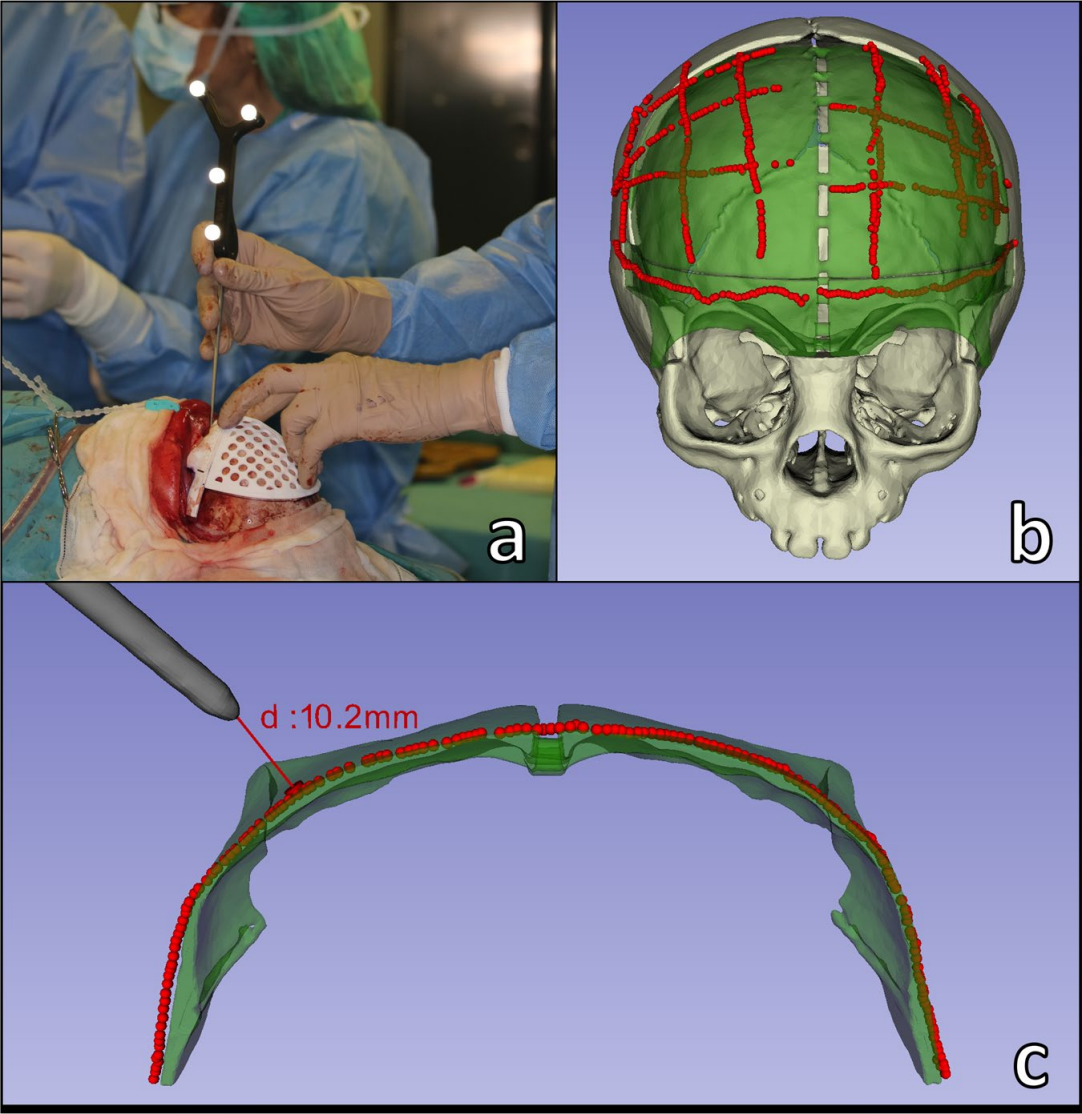
(**a**) Surgeon recording registration points on 3D printed osteotomy guides using the tracked pointer tool; (**b**) Navigation points recorded on remodeled bone surface (red) and virtual surgical planning (green) [Supplementary Video 1–3]; (**c**) Navigation on supraorbital bar region using tracker pointer tool [Supplementary Video 4].

**Figure 5:**
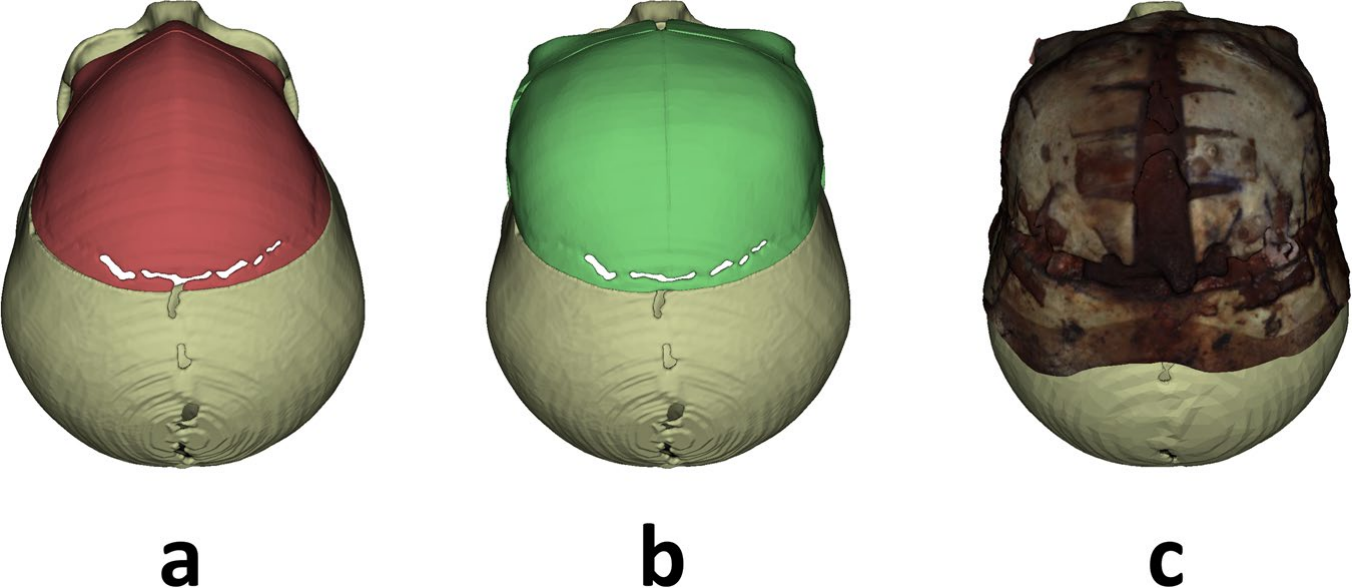
Superior view of (**a**) preoperative skull model, (**b**) virtual surgical planning and (**c**) postoperative bone surface scan.

The standard procedure for fronto-orbital advancement to treat metopic and unicoronal synostoses involves repositioning of the frontal bones and supraorbital bar^26^. Therefore, for the clinical cases evaluated in this study, surgical planning included osteotomies of the frontal bones and the supraorbital bar. Once the preoperative 3D virtual model of the skull was available, the surgeons performed virtual surgery on a computer workstation with the collaboration of biomedical engineers. Specific CAD software (Freeform® Plus) was used for the following: (1) planning the surgical osteotomies (Fig. 2b), (2) creating 3D models of patient-specific surgical cutting guides to assist during intraoperative osteotomies and including reference points for intraoperative navigation (Fig. 2c), (3) simulating affected bone remodeling (Fig. 2d,e), (4) generating 3D models of templates to assist during intraoperative bone remodeling (Fig. 2e), and (5) generating 3D models of the skull using the virtually remodeled bone fragments. All virtual models were exported in stereolithography (STL) file format.

Once the design was concluded, surgical guides and templates were manufactured using selective laser sintering (additive manufacturing) and polyamide material (KLS Martin Group, Tuttlingen, Germany). All 3D printed models were sterilized before surgery using standard autoclave protocols.

### Intraoperative navigation

A software application called *CranioNav* was specifically developed as a module in the 3D Slicer platform^25,27^ to assist surgeons during the procedures. *CranioNav* enables the importation of CT imaging studies, anatomical 3D models, and preoperative virtual planning.

A Polaris Spectra (NDI, Waterloo, Canada) optical tracking system was used for real-time tool positioning. The device was mounted on a tripod inside the operating room, and its position was adjusted to ensure that the field of view covered the surgical area. The optical tracker includes two infrared cameras and enables the tracking of instruments containing a configuration of three or more reflective optical markers. The IGS system uses a pointer tool composed of four spherical optical markers, which is sterilized and used by the surgeon during navigation. Positioning data from the tracking device are transferred to *CranioNav* using the PLUS toolkit^28^ and the OpenIGTLink communication protocol^29^.

Preoperative image data and the intraoperative physical anatomy were aligned using a two-step registration procedure based on patient-specific 3D printed surgical guides and external markers attached intraoperatively. This registration technique did not require the patient’s head to be immobilized during surgery or markers to be attached to the patient’s anatomy prior to CT imaging. During the first step, once osteotomy guides were fitted to the patient’s bone surface, predefined points of the 3D printed guides were recorded with the tracked pointer tool (Fig. 3a). A *primary registration* was obtained using these landmarks. Then, a *secondary registration* was performed by recording the position of external markers attached intraoperatively to the bone (Fig. 3b). The markers used were resorbable pins (SonicPins Rx, KLS Martin, Tuttlingen, Germany), which were inserted by drilling holes in the bone tissue surrounding the affected region. This two-step registration makes it possible to account for the position of the head since the secondary registration can be repeated at any time during the procedure.

*CranioNav* was displayed on a screen adjacent to the surgical field in order to guide the surgeons during the intraoperative navigation steps. First, it enabled the user to record registration points using the tracked pointer tool and to perform intraoperative registration. The software displayed registration errors and enabled the user to repeat the registration procedure when needed. Once registration had been performed, *CranioNav* displayed the real-time position of the tracked pointer with respect to the CT imaging data and virtual anatomical 3D models of the patient. The application enabled the surgeon to visualize different 3D views of the scene, which can be defined preoperatively. Moreover, the system displayed the real-time distance from the tracked pointer tool tip to virtual planning and anatomical regions of interest, thus providing visual and acoustic feedback to the surgeon (Fig. 4c). It is also possible to record point coordinates and perform geometrical measurements, which may be clinically relevant for the surgeon.

### Surgical procedure

A bicoronal S-shaped incision was made to expose the frontal and supraorbital regions. Once the bone surface was exposed, 3D printed patient-specific surgical guides were placed on the cranium, and the six resorbable registration pins were attached to the surface of the healthy bone outside the osteotomy region. Then, *primary registration* was performed by recording the position of six predefined characteristic points included in the surgical guides using the tracked pointer tool (Fig. 4a). After primary registration, the positions of the resorbable pins were recorded for later usage during the *secondary registration* procedure.

Osteotomies of the frontal bone and supraorbital bar were performed following the edges of the cutting guides. The affected bone tissue was removed and then reshaped using the 3D printed patient-specific templates. Resorbable plates and pins were used to reinforce and reshape the bone tissue.

The next step was the placement and fixation of the remodeled bone over the exposed dura. First, secondary registration was performed by recording the position of the resorbable registration pins. Then, *CranioNav* displayed the position of the tracked pointer tool tip with respect to virtual anatomical models and CT images of the patient. The IGS system enabled the surgeon to verify that the reshaped bone has been placed in the location defined during preoperative virtual planning by recording the bone surface using the tracked pointer tool (Fig. 4b,c). The final position was obtained after an iterative process consisting of: (1) partially fixing the remodeled bone fragment to surrounding healthy bone using resorbable plates and screws, (2) recording bone fragment position using tracked pointer tool, (3) comparing recorded position with virtual surgical planning, and (4) making corrections to match target position defined in planning based on the metrics provided by the navigation software and the surgeon’s visual assessment. The patient’s head was not immobilized during surgery, with the result that secondary registration was frequently repeated to minimize navigation error.

Once the optimal position had been achieved, the remodeled bone tissue was stabilized in place using extra resorbable plates and screws. The final surgical outcome was recorded by moving the tracked pointer tool along the surface of the reconstructed supraorbital bar and frontal bone.

### Intraoperative surface scanning

Once remodeled bone fragments were positioned and fixed, intraoperative 3D surface scanning was performed to document and evaluate the outcome of cranial vault reconstruction using Artec EVA® structured light scanner (Artec Group, Luxembourg) (Fig. 5). Preoperative and postoperative scans were performed for each surgery. This hand-held scanning device illuminates the surgical area with striped-patterns of bright white light and computes a 3D surface mesh from the deformation of the patterns. In addition, a third camera is used to obtain color texture information. During the scanning process, this device was moved around the region of interest, and the acquired 3D images were automatically aligned and fused using geometric and textural information (Supplementary Video 5). The final 3D surface, texture, and mapping information were exported in Wavefront OBJ (.obj), Joint Photographic Experts Group (.jpg), and Material Template Library (.mtl) file formats, respectively.

### Data analysis and evaluation

In order to evaluate the accuracy of intraoperative registration performed during the five navigated craniosynostosis reconstruction surgeries, the root-mean-squared error (RMSE) was computed at the fiducials for all repetitions of primary and secondary registrations. The time spent on this task was measured.

The accuracy of the navigation system was estimated by recording points along the surface of the remodeled bone. Intraoperative surface scanning data were used as the gold-standard to measure navigation error. The intraoperatively scanned bone surface was registered to the navigation data using the position of the resorbable pins used for secondary registration. These pins can be identified in the surface scan thanks to texture information provided by the scanning device. Therefore, a point-based registration can be performed to align the surface scan with the navigation data. After registration, the navigation error was estimated as the average absolute distance between the recorded navigation points and the intraoperatively scanned surface.

Finally, the surgical outcome of fronto-orbital advancement was evaluated by measuring the interfrontal angle and transverse forehead width. These metrics were computed using three landmarks, one located at the left frontal bone (LFL), one at the right frontal bone (RFL) and one along the metopic suture (MSL). These landmarks were manually selected for each subject. Interfrontal angle is defined as the angle formed by the left frontal segment (LFL-MSL) and right frontal segment (RFL-MSL) and transverse forehead width value is measured as the distance between the two most lateral landmarks (LFL and RFL). Both metrics were computed in the preoperative model, in the virtual surgical plan, and in the intraoperative surface scan. Surgical outcome error was estimated as the difference between the virtual planned and the postoperative values.

## Results

Primary registration, which was performed using six landmarks on the 3D printed surgical guides, yielded an average RMSE of 0.94 ± 0.27 mm for the five surgeries under study. The average time required to perform the registrations was 50 ± 10 seconds. Secondary registration, which was performed using the position of six resorbable pins attached intraoperatively, yielded an average RMSE of 1.30 ± 0.47 mm. Of note, this secondary registration was performed 17, 9, 12, 9 and 9 times for surgeries 1, 2, 3, 4 and 5, respectively. This registration step must be repeated to avoid increased navigation error caused by the patient’s head movement. The average duration of secondary registrations was 40 ± 14 seconds. The results are summarized in Table 1.

**Table 1:** Root mean squared error and duration of primary and secondary registrations.

Patient	Intraoperative Registration			
	Primary		Secondary	
	RMSE (mm)	Duration (s)	RMSE (mm)	Duration (s)
1	0.65	42	1.87 ± 0.31	32.41 ± 9.17
2	1.07	65	1.16 ± 0.34	57.56 ± 5.31
3	1.25	55	0.96 ± 0.14	34.00 ± 8.13
4	0.58	54	1.19 ± 0.09	36.60 ± 5.00
5	1.18	36	0.90 ± 0.32	49.56 ± 14.84
Avg.	0.94 ± 0.27	50.40 ± 10.25	1.30 ± 0.47	40.50 ± 13.61

**Figure 6:**
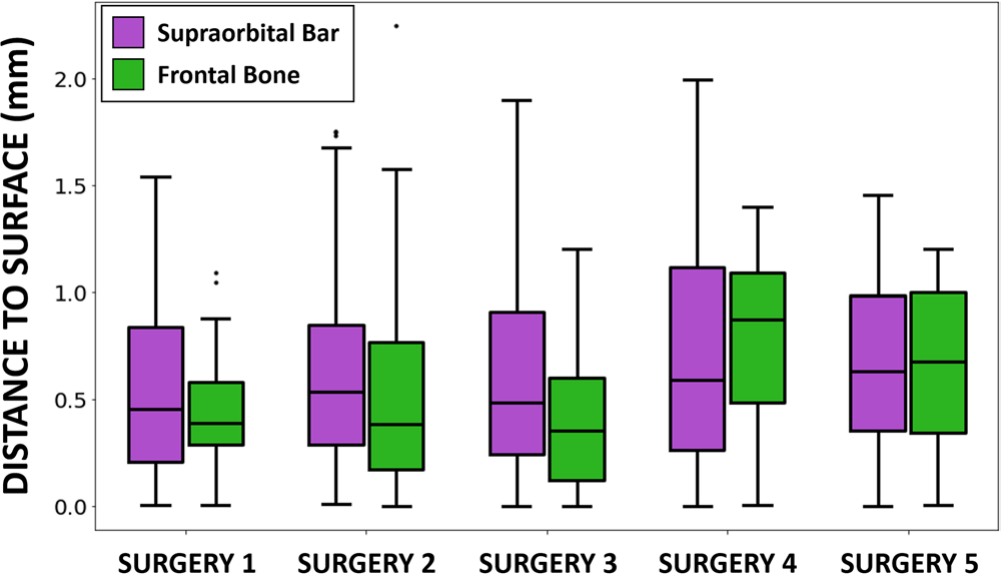
Estimated navigation error using the intraoperative surface scan as the gold-standard. Error is computed as the point-to-surface distance between the points recorded during the navigation and the cranial vault surface reconstructed intraoperatively using Artec Eva structured light scanner.

The results of the estimation of navigation accuracy using intraoperative scanning as a gold-standard are shown in Fig. 6. An average error of 0.63 mm ± 0.42 mm was obtained. The error was 0.62 mm ± 0.39 mm in the remodeled frontal region and 0.64 mm ± 0.46 mm in the remodeled supraorbital bar. The statistical analysis of the error distribution data from Fig. 6 shows that 75% of the recorded points (third quartile) present an error below 0.97 mm.

Interfrontal angle and transverse forehead width measurements are shown in Table 2. Preoperative values correlate with the bitemporal narrowing caused by metopic synostosis and unilateral supraorbital recession associated with unicoronal synostosis. The absolute difference between target and postoperative interfrontal angles was 6.31°, 0.48°, 1.05°, 5.38° and 4.91° for surgery 1, 2, 3, 4 and 5, respectively. The average error was 3.63°. Transverse forehead width absolute error was 3.27, 1.12, 1.68, 3.30 and 0.06 mm for surgery 1, 2, 3, 4 and 5, respectively. The average error was 1.89 mm. This difference with respect to virtual surgical planning is caused mainly by the flexibility of the remodeled bone, which is composed of several fragments fixed with resorbable plates and screws. In addition, surgeons usually make slight modifications to virtual planning based on their experience. As a general rule, increased overcorrection is applied in surgical planning, but the degree of overcorrection is usually reduced intraoperatively owing to practical limitations such as skin elasticity. This pattern can be observed in the interfrontal angle and transverse forehead width results, since measured postoperative values were lower than target values in all five patients.

**Table 2:** Interfrontal angle and transverse forehead width measured in preoperative skull model (pre-op), virtual surgical plan (VSP) and postoperative surface scan (post-op).

ID	Type	Interfrontal Angle (°)			Transverse Forehead Width (mm)		
		Pre-Op	VSP	Post-Op	Pre-Op	VSP	Post-Op
1	Metopic	99.70	130.03	123.72	78.57	93.28	90.01
2	Metopic	99.41	135.14	134.66	73.78	88.41	87.29
3	Unicoronal	125.52	144.21	143.17	93.07	97.83	96.15
4	Metopic	112.24	130.38	125.00	88.51	95.46	92.16
5	Metopic	116.94	139.38	134.46	78.53	84.65	84.60

**Figure 7:**
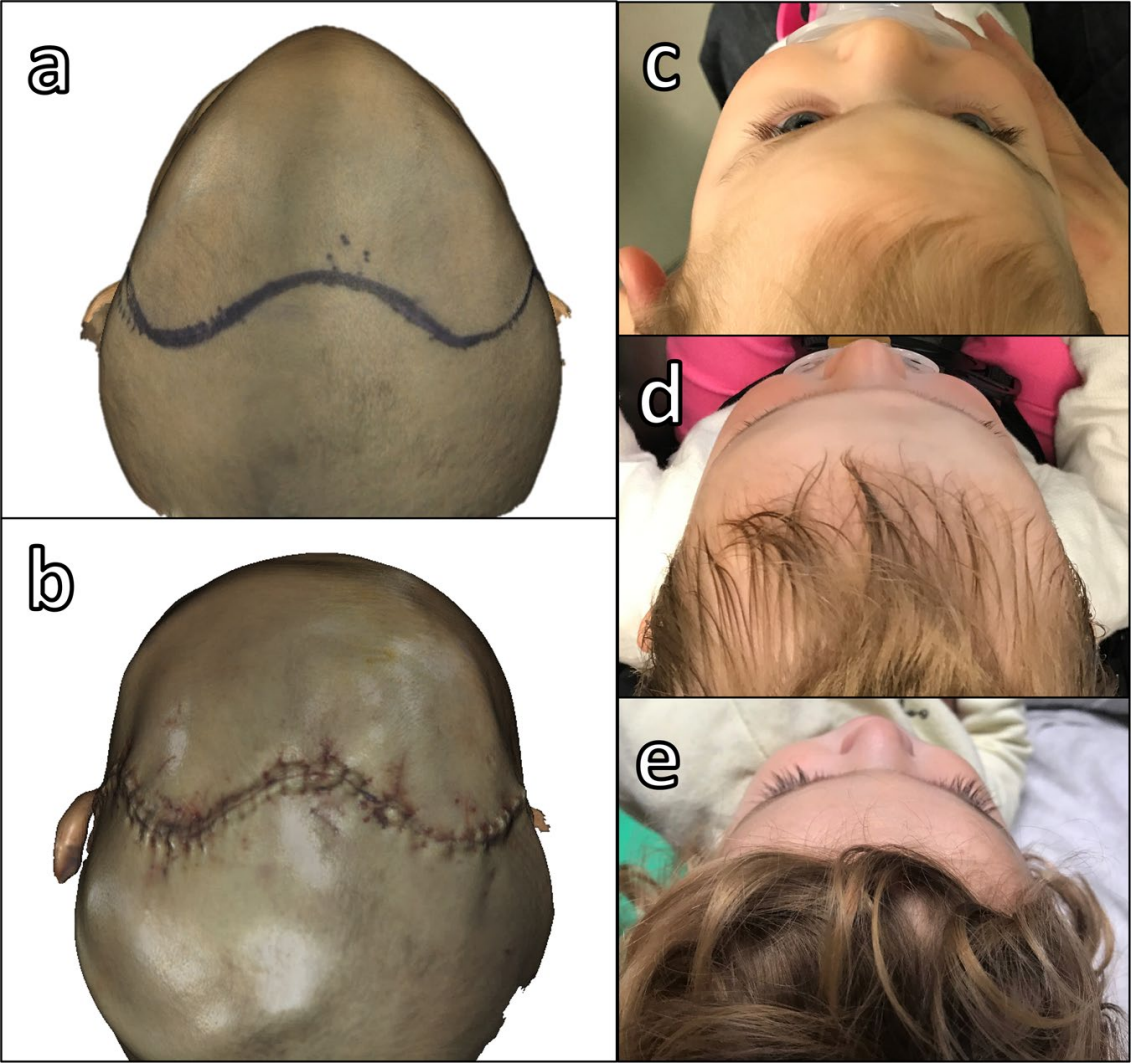
Patient 1: (**a**) Intraoperative surface scan before remodeling, (**b**) Intraoperative surface scan after remodeling, (**c**) Photograph before surgery, (**d**) Photograph 4 months after surgery, and (**e**) Photograph 1 year after surgery.

Surgical outcome for patients presenting trigonocephaly shows improved bitemporal width and correction of metopic ridging. Correction of plagiocephaly improved orbital symmetry and forehead projection. Symmetry, harmony, and balance between the face and cranial vault were achieved in all five patients (Fig. 7).

## Discussion and Conclusion

In this study, we propose a novel methodology for the surgical correction of craniosynostosis. The workflow is based on the use of intraoperative navigation combined with patient-specific 3D printed guides and templates. The method was evaluated in the cranial vault reconstruction procedures of five patients with craniosynostosis and yielded optimal matching with preoperative virtual planning.

Our intraoperative navigation solution does not require the patient’s head to be immobilized or external markers to be attached prior to surgery. The proposed two-step registration procedure uses 3D printed surgical guides and intraoperatively attached markers as landmarks. This methodology enables the user to repeat the registration multiple times during surgery to minimize navigation error. The average time spent during the primary and secondary registration steps was 50 and 40 seconds, respectively. Therefore, intraoperative registration does not substantially increase surgical time and it is a good alternative to head immobilization in this kind of procedures.

The navigation system provides valuable information to the surgeon for accurate bone fragment positioning. *CranioNav* displays real-time tool position with respect to 3D models and preoperative CT images. The position of remodeled bone tissue can be modified according to navigation data to ensure accurate matching with preoperative virtual planning. The available functionalities, such as real-time tool-to-surface distance or reconstructed bone outline recording, facilitate the surgeons’ work. Navigation error was evaluated using intraoperative surface scanning as a reference. An average error of <0.7 mm was obtained, thus indicating the accuracy of real-time tool positioning and intraoperative registration achieved by the system. Surgeons were trained to use the navigation system by performing simulations on phantoms prior to surgeries. Our system presented a short learning curve, and surgeons were able to achieve good performance after several simulations.

The portable scanning device enables non-invasive, radiation-free, anesthetic-free and fast acquisition of 3D photographs of the reconstructed cranial vault. This technology could replace postoperative CT imaging studies for outcome and follow-up analysis, thus avoiding exposure to unnecessary radiation. Moreover, the portability of this device enables its use in the operating room for intraoperative reconstruction and outcome estimation. Intraoperative scans could be directly compared with the preoperative virtual plan to assess the quality of the surgical outcome.

Surgical outcome was evaluated by measuring the interfrontal angle and transverse forehead width in the preoperative skull model, virtual surgical plan, and intraoperative surface scan. The average difference between the interfrontal angle and transverse forehead width defined during planning and the values achieved after surgical reconstruction was 3.63° and 1.89 mm, respectively. Moreover, the postoperative results show symmetry, harmony, and balance between the face and cranial vault in all five patients. The use of normative skull models as reference during virtual planning could improve surgical outcomes and reduce inter-surgeon variability in the determination of the target cranial vault shape. Although normative age-matched skull models were not used in this study, their use would not require any modifications in our workflow.

This novel workflow for craniosynostosis reconstruction based on intraoperative navigation and 3D printing enables an accurate translation of preoperative surgical planning into the operating room, thus improving the reproducibility of craniosynostosis surgeries and reducing inter-surgeon variability. Therefore, interventional plans minimizing cranium malformations can be used in actual surgeries to ensure optimal outcomes without a substantial increase in surgical time.

## Supplementary information

Supplementary information file

Supplementary Video 1

Supplementary Video 2

Supplementary Video 3

Supplementary Video 4

Supplementary Video 5

## Data Availability

The datasets generated and/or analyzed during the current study are available from the corresponding author on reasonable request.
