# Supplementary material for: Craniosynostosis surgery: workflow based on virtual surgical planning, intraoperative navigation and 3D printed patient-specific guides and templates
Source: Sci Rep. 2019 Nov 27;9:17691. doi: 10.1038/s41598-019-54148-4 (PMC6881390; doi:10.1038/s41598-019-54148-4)
Supplement: Supplementary file 1 — Supplementary information file [file 41598_2019_54148_MOESM1_ESM.pdf]

Title: "Craniosynostosis surgery: workflow based on virtual surgical planning, intraoperative navigation and 3D printed patient-specific guides and templates"

Author list: David García-Mato, MSc[1,2]; Santiago Ochandiano, MD DMD PhD[2,3]; Mónica García-Sevilla, MSc[1,2]; Carlos Navarro-Cuéllar, MD DMD PhD[2,3]; Juan V. Darriba-Allés, MD[2,4]; Roberto García-Leal, MD[2,4]; José A. Calvo, MD PhD[2,5]; Rubén Pérez-Mañanes, MD PhD[2,5]; José I. Salmerón, MD PhD[2,3], *Javier Pascau, MSc PhD[1,2]

1. Departamento de Bioingeniería e Ingeniería Aeroespacial, Universidad Carlos III de Madrid, Madrid, Spain
2. Instituto de Investigación Sanitaria Gregorio Marañón, Madrid, Spain
3. Servicio de Cirugía Oral y Maxilofacial, Hospital General Universitario Gregorio Marañón, Madrid, Spain
4. Servicio de Neurocirugía, Hospital General Universitario Gregorio Marañón, Madrid, Spain
5. Servicio de Cirugía Ortopédica y Traumatología, Hospital General Universitario Gregorio Marañón, Madrid, Spain

Contact information:

David García-Mato, MSc (dgmato@ing.uc3m.es); Santiago Ochandiano, MD DMD PhD (sochandiano@hotmail.com); Mónica García-Sevilla, MSc (mongarci@pa.uc3m.es); Carlos Navarro-Cuéllar, MD DMD PhD (cnavarrocuellar@gmail.com); Juan V. Darriba-Allés, MD (juanvidarriba@gmail.com); Roberto García-Leal, MD (rgarcia_leal@yahoo.es); José A. Calvo, MD PhD (calvoharo@yahoo.es); Rubén Pérez-Mañanes, MD PhD (rubenperez.phd@gmail.com); José I. Salmerón, MD PhD (jisalmeron@telefonica.net); *Javier Pascau, MSc PhD (jpascau@ing.uc3m.es)

Corresponding author:

Javier Pascau, MSc PhD

Avenida de la Universidad 30

28911 Leganés, Madrid, Spain

jpascau@ing.uc3m.es

SUPPLEMENTAL DIGITAL CONTENT

Supplemental digital content has been submitted in conjunction with the article:

- Supplementary video 1: Intraoperative navigation using tracked pointer tool. The surgeon moves the tool and records points along the surface of the remodeled supraorbital bar to verify and correct its position. Recorded points (red) can be visualized on the navigation screen with respect to preoperative virtual surgical plan (green).

- Supplementary video 2: Intraoperative navigation using tracked pointer tool. The surgeon verifies remodeled bone position by looking at the navigation screen. The tracked pointer tool is moved along the remodeled supraorbital bar while the navigation software provides real-time feedback of the distance to the target position (virtual surgical plan).

- Supplementary video 3: Intraoperative navigation using tracked pointer tool. The surgeon moves the tool and records points along the surface of the remodeled supraorbital bar to verify and correct its position. Recorded points (red) can be visualized on the navigation screen with respect to preoperative virtual surgical plan (green).

- Supplementary video 4: Intraoperative navigation using tracked pointer tool. The surgeon moves the tool and records points along the surface of the remodeled frontal region to verify and correct its position. Recorded points (blue) can be visualized on the navigation screen with respect to preoperative virtual surgical plan (green).

- Supplementary video 5: Intraoperative surface scanning using Artec Eva structured light scanner. The device is moved around the surgical area at a distance range of 0.4-1 meters for 3D frame acquisition. This hand-held device illuminates the surgical area with stripped-patterns of bright white light and computes a 3D surface mesh from the deformation of these patterns. In addition, a third camera is used to obtain color texture information. A 3D model of the cranial vault is generated by aligning and fusing the acquired 3D images using geometric and textural information.
